# Dual Antiplatelet Therapy Duration Following Percutaneous Coronary Intervention: Time for a Change

**DOI:** 10.1016/j.jscai.2024.102510

**Published:** 2025-02-18

**Authors:** Dean J. Kereiakes

**Affiliations:** a The Christ Hospital Heart and Vascular Institute, Cincinnati, Ohio; b Lindner Center for Research and Education, Cincinnati, Ohio

**Keywords:** dual antiplatelet therapy, percutaneous coronary intervention

Following percutaneous coronary intervention (PCI), dual antiplatelet therapy (DAPT) with aspirin and a P2Y12 inhibitor (P2Y12i) is administered to reduce atherothrombotic events involving both stented and nonstented coronary segments.^1^ Current clinical practice guidelines (CPG) recommend DAPT durations of 6 months following PCI for chronic coronary syndromes and 12 months for patients with acute coronary syndromes (ACS), although “select patients may be transitioned to P2Y12i monotherapy following 1 to 3 months of DAPT after consideration of ischemic and bleeding risks.”^1^^,^^2^

The optimal duration for DAPT prescription has been based on an individual patient’s perceived relative risk of ischemic vs bleeding events. Multiple risk scores have been proposed to stratify relative risks and to inform the duration as well as intensity of DAPT.^3^^,^^4^ Recent randomized controlled trials and meta-analyses have suggested the following: (1) short (1-3 months) DAPT followed by platelet inhibitor monotherapy (aspirin or P2Y12i) is best prescribed to patients at greatest bleeding risk (Class IIa CPG indication)^1^^,^^5^; (2) P2Y12i (vs aspirin) monotherapy following DAPT is associated with net clinical benefit (lower bleeding and ischemic event rates)^6^, ^7^, ^8^; and (3) DAPT deescalation (transition from prasugrel/ticagrelor to clopidogrel or half dose prasugrel/ticagrelor) is best prescribed to patients at greatest ischemic risk (Class IIb CPG indication).^1^

A recent network meta-analysis suggests that unguided DAPT deescalation provides the safest, most effective strategy for reducing major adverse cardiovascular ischemic events as well as major/minor bleeding^9^ and that short DAPT is the best strategy for reducing major bleeding and all-cause death. Subgroup analyses (ACS, high bleeding risk, complex PCI) suggest that both short DAPT and DAPT deescalation strategies reduce major bleeding without increasing ischemic events when compared with standard DAPT durations regardless of clinical presentation or procedural complexity.^10^^,^^11^ Importantly, no “one size fits all-comers” strategy for DAPT after PCI has been proposed.

In this context, Dodoo et al^12^ have performed a network meta-analysis (7 randomized controlled trials; 34,774 patients) published in this issue of *JSCAI* that aimed to assess the clinical utility of ultrashort (≤1 month) DAPT (US-DAPT) followed by P2Y12i monotherapy compared with standard (≥6 months) DAPT duration after PCI. Furthermore, the relative safety and effectiveness of clopidogrel vs ticagrelor monotherapy following US-DAPT was analyzed. The study population included those with ST-segment elevation myocardial infarction (STEMI; 15.9%), non-STEMI (19.5%), unstable angina (16.8%), and chronic coronary syndromes (47.8%). The authors conclude as follows: US-DAPT followed by P2Y12i monotherapy is associated with lower rates of net adverse clinical events and bleeding without increasing the rate of ischemic events when compared with standard DAPT duration, regardless of P2Y12i choice (ticagrelor or clopidogrel).

This study focuses on current confusion regarding individualized DAPT prescription and suggests the “best” DAPT strategy to be short DAPT followed by P2Y12i monotherapy with specific caveats. First, the Short and Optimal duration of Dual Anti-platelet Therapy-2 study for patients with ACS trial randomly assigned East Asian patients undergoing PCI for ACS to receive either 1 to 2 months DAPT followed by clopidogrel monotherapy or DAPT for 12 months.^13^ The study's primary end point (composite of cardiac death, MI, definite stent thrombosis, stroke, TIMI major/minor bleeding) powered for noninferiority of short DAPT was not achieved. A reduction in major bleeding observed with short DAPT was counterbalanced by an increase in ischemic events. Of note, this study enrolled an East Asian population (increased cytochrome P450 [CYP] 2C19 polymorphisms and clopidogrel response variability) with ACS (increased ischemic risk).

The Short and Optimal duration of Dual Anti-platelet Therapy-2 study for patients with ACS trial differs from other studies demonstrating no difference in ischemic events and a significant decrease in bleeding among patients assigned to short (1-3 months) ticagrelor-based DAPT followed by ticagrelor monotherapy compared with longer DAPT durations.^10^^,^^14^ Second, the potency and consistency of platelet inhibition as well as the time course following PCI may influence the choice of P2Y12i in an “at-risk” (East Asian, ACS) population. In this regard, concern regarding clopidogrel monotherapy relates to the association between high residual “on-treatment” platelet reactivity and adverse clinical outcomes among subjects with clopidogrel response variability related to genotype (CYP2C19 polymorphisms) and/or phenotype (body mass index, renal function, diabetes mellitus). In these subjects, longer DAPT duration (≥3 months) prior to clopidogrel monotherapy may be beneficial. For example, the Harmonizing Optimal Strategy for Treatment of coronary artery stenosis- Extended Antiplatelet Monotherapy trial compared clopidogrel to aspirin monotherapy in the chronic maintenance period (6-18 months) after PCI.^11^^,^^15^ Despite enrollment of East Asians with ACS, clopidogrel provided net clinical benefit driven by reductions in both ischemic and bleeding end points. Adverse events were infrequent late (6-18 months) after PCI, which suggests that clopidogrel response variability at later time points (beyond 1-2 months), when endoluminal disruption by ACS/PCI has been stabilized by additional guideline-driven adjunctive pharmacotherapies, may not be associated with increased ischemic events.

Conversely, early (1-2 months) use of more potent and consistent P2Y12i agents with less interindividual clopidogrel response variability (ticagrelor/prasugrel) following PCI for ACS appears desirable as evidenced by results of the Ticagrelor Monotherapy in Patients with new-generation Drug-Eluting Stents for Acute Coronary Syndrome trial. In the Ticagrelor Monotherapy in Patients with new-generation Drug-Eluting Stents for Acute Coronary Syndrome trial, East Asian patients with ACS (40% STEMI) underwent PCI and were randomly assigned to receive ticagrelor monotherapy after ≤1-month DAPT (median 16 days) or ticagrelor-based DAPT. The primary end point was a net clinical benefit (composite of all-death MI, definite/probable stent thrombosis, stroke, and major bleeding) at 1 year after the index PCI. Major bleeding was significantly reduced, ischemic events were numerically reduced, and net clinical benefit favored the ticagrelor monotherapy arm (HR, 0.54; 95% CI, 0.37-0.80; *P*_noninferiority_ < .001; *P*_superiority_ = .002) despite the at-risk (East Asian, ACS) study population. Finally, the longer-term benefit of platelet inhibition following PCI for ACS may reflect secondary prevention of atherothrombotic events not related to stented coronary segments.

The Dual AntiPlatelet Therapy trial randomly assigned subjects who were bleeding and ischemic event–free at 1 year after PCI to receive either continued DAPT or aspirin monotherapy. More than half of all the MI prevented by DAPT (enhanced platelet inhibition) were not related to stented coronary segments.^16^ Recent analyses involving longer-term platelet inhibition in subjects with coronary disease suggest that P2Y12i monotherapy (either ticagrelor or clopidogrel) provides greater safety and effectiveness than aspirin.^7^

In summary, Dodoo et al^12^ confirm and extend evidence suggesting that short (1-3 months) DAPT followed by P2Y12i monotherapy represents the optimal DAPT strategy for all-comers undergoing PCI regardless of clinical presentation or procedural complexity; longer (3 months vs 1 month) DAPT with more potent/consistent P2Y12i (ticagrelor or clopidogrel) is best for “at-risk” populations; longer-term P2Y12i monotherapy (either clopidogrel or ticagrelor) provides greater safety and effectiveness than aspirin monotherapy, and finally, current CPG should be revised to elevate short (1-3 months) DAPT durations tailored for clinical acuity followed by P2Y12i monotherapy. Further study is required to directly compare short DAPT vs DAPT deescalation strategies, although both strategies reduce bleeding risk without increasing ischemic hazard when compared with current CPG-recommended DAPT durations following PCI.
